# Determination of GC content of *Thermotoga maritima, Thermotoga neapolitana and Thermotoga thermarum* strains: A GC dataset for higher level hierarchical classification

**DOI:** 10.1016/j.dib.2016.05.045

**Published:** 2016-05-27

**Authors:** Bhagwan N. Rekadwad, Chandrahasya N. Khobragade

**Affiliations:** School of Life Sciences, Swami Ramanand Teerth Marathwada University, Nanded, Maharashtra 431606, India

**Keywords:** ENDMEMO, GC content, Hyperthermophiles, New digital data, Whole genome

## Abstract

A total of 16 strains of hyperthermophilic *Thermotoga* complete genome sequences viz. *Thermotoga maritima* (AE000512, CP004077, CP007013, CP011107, NC_000853, NC_021214, NC_023151, NZ_CP011107, CP011108, NZ_CP011108, CP010967 & NZ_CP010967), *Thermotoga neapolitana* (CP000916, & NC_011978) and *Thermotoga thermarum* (CP002351 & NC_015707) complete genome sequences were retrieved from NCBI BioSample database. ENDMEMO GC used for creation of data on GC content in *Thermotoga* sp. DNA sequences. Maximum GC content was observed in *Thermotoga* strains AE000512 & NC_000853 (69 %GC), followed by NZ_CP011108, CP011108, NZ_CP011107, NC_023151, NC_021214, CP011107 & CP004077 (68.5 %GC), followed by NZ_CP010967 & CP010967 (68.3 %GC), followed by CP000916, CP007013 & NC_011978 (68 %GC), followed by CP002351 & NC_015707 (67 %GC) strains. The use of GC dataset ratios helps in higher level hierarchical classification in Bacterial Systematics in addition to phenotypic and other genotypic characters.

Specifications Table

**Table:** 

Subject area	*Life Sciences*
More specific subject area	*Extremophiles, Microbiology, Bacterial Systematics, Bioinformatics*
Type of data	*Table, graphical representations*
How data was acquired	*Through NCBI BioSample database*
Data format	*Raw and Analyzed*
Experimental factors	*GC content were determined using bioinformatics tool*
Experimental features	*Complete genome sequences were used*
Data source location	Bioinformatics Research Laboratory, School of Life Sciences, S. R. T. M. University, Nanded, India
Data accessibility	Data available within article and via the *NCBI repository*http://www.ncbi.nlm.nih.gov/nuccore.

Value of the data

• Data provides information of the GC content of hyperthermophilic Thermotoga species.
• This data would be valuable for quantitative analysis of newly isolated *Thermotoga maritima, Thermotoga neapolitana* and *Thermotoga thermarum* strains.
• This data would be valuable for higher level hierarchical classification of Thermotoga strains in Bacterial Systematics.

## 1 Data

This paper contains data on GC percentage of 16 strains of hyperthermophilic Thermotoga. Complete genome sequences of *Thermotoga maritima* (AE000512, CP004077, CP007013, CP011107, NC_000853, NC_021214, NC_023151, NZ_CP011107, CP011108, NZ_CP011108, CP010967 & NZ_CP010967), *Thermotoga neapolitana* (CP000916, & NC_011978) and *Thermotoga thermarum* (CP002351 & NC_015707) were downloaded from NCBI nuccore. Maximum, minimum and average GC percent of 16 *Thermotoga* strains were determined and digitised using ENDMEMO GC calculating and GC plotting tool. See also *NCBI repository* http://www.ncbi.nlm.nih.gov/nuccore.

## 2 Experimental design, materials and methods

Complete genome sequences of *Thermotoga maritima* (AE000512, CP004077, CP007013, CP011107, NC_000853, NC_021214, NC_023151, NZ_CP011107, CP011108, NZ_CP011108, CP010967 & NZ_CP010967), *Thermotoga neapolitana* (CP000916, & NC_011978) and *Thermotoga thermarum* (CP002351 & NC_015707) strains were downloaded from NCBI nuccore in FASTA format. Using ENDMEMO GC calculating and GC plotting tool was used to determine the exact minimum, maximum and average GC in percent in the complete genome sequence of 16 *Thermotoga* strains (Table 1). ENDMEMO GC plotter showed a pattern of GC distribution in complete DNA sequence showed through graphical representations in Figs. 1–16 (See supplementary Figs. 1–16). Upper and lower red line indicate maximum and minimum percentage of GC content distribution in complete DNA sequence, while middle blue line indicates average GC percentage [1], [2], [3], [4], [5].

## Legends to Figs. 1–16

Figs. 1–16 GC percentage in complete DNA sequences of hyperthermophile *Thermotoga* strains (AE000512, CP004077, CP007013, CP011107, NC_000853, NC_021214, NC_023151, NZ_CP011107, CP011108, NZ_CP011108, CP010967, NZ_CP010967, CP000916, NC_011978, CP002351 and NC_015707).

## Figures and Tables

**Table 1:** GC percentage in complete DNA sequences of hyper-thermophile *Thermotoga* strains.

**SN**	**Species**	**Accession numbers**	**Maximum GC %**	**Minimum GC %**	**Average GC %**	**Supplementary Figs.**
s1	*Thermotoga maritima* MSB8	AE000512	69	21.5	46.2	Fig. 1
2	*Thermotoga maritima* MSB8	CP004077	68.5	21	46.2	Fig. 2
3	*Thermotoga maritima* MSB8	CP007013	68	23	46.2	Fig. 3
4	*Thermotoga maritima* MSB8	CP011107	68.5	21	46.3	Fig. 4
5	*Thermotoga maritima* MSB8	NC_000853	69	21	46.2	Fig. 5
6	*Thermotoga maritima* MSB8	NC_021214	68.5	21	46.2	Fig. 6
7	*Thermotoga maritima* MSB8	NC_023151	68.5	21	46.2	Fig. 7
8	*Thermotoga maritima* MSB8	NZ_CP011107	68.5	21	46.3	Fig. 8
9	*Thermotoga maritima* strain Tma100	CP011108	68.5	21	46.2	Fig. 9
10	*Thermotoga maritima* strain Tma100	NZ_CP011108	68.5	21	46.2	Fig. 10
11	*Thermotoga maritima* strain Tma200	CP010967	68.3	21	46.3	Fig. 11
12	*Thermotoga maritima* strain Tma200	NZ_CP010967	68.3	21	46.3	Fig. 12
13	*Thermotoga neapolitana* DSM 4359	CP000916	68	25	46.5	Fig. 13
*14*	*Thermotoga neapolitana DSM 4359*	NC_011978	68	25	46.9	Fig. 14
15	*Thermotoga thermarum* DSM 5069	CP002351	67	23.5	40.3	Fig. 15
16	*Thermotoga thermarum* DSM 5069	NC_015707	67	23.5	40.3	Fig. 16

## Footnotes

Transparency document Transparency data associated with this article can be found in the online version at 10.1016/j.dib.2016.05.045.
