# Supplementary figures and images for: Determination of GC content of Thermotoga maritima, Thermotoga neapolitana and Thermotoga thermarum strains: A GC dataset for higher level hierarchical classification
Source: Data Brief. 2016 May 27;8:300–3. doi: 10.1016/j.dib.2016.05.045 (PMC4906128; doi:10.1016/j.dib.2016.05.045)

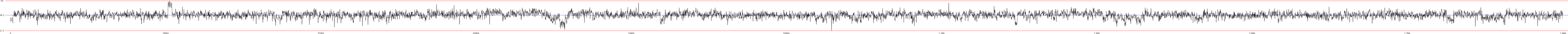

Supplement: Supplementary file 1 — Supplementary material [file mmc1.zip › mmc1.png]

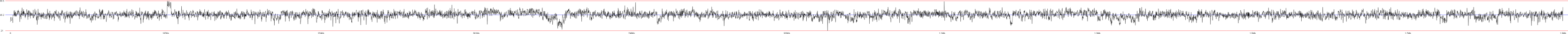

Supplement: Supplementary file 2 — Supplementary material [file mmc2.zip › mmc2.png]

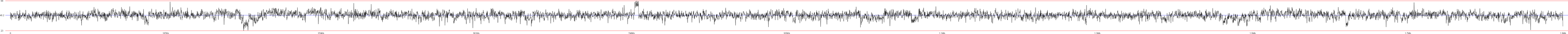

Supplement: Supplementary file 3 — Supplementary material [file mmc3.zip › mmc3.png]

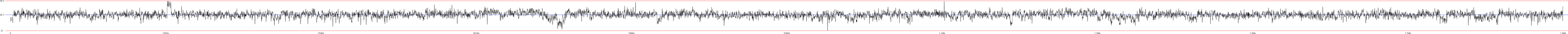

Supplement: Supplementary file 4 — Supplementary material [file mmc4.zip › mmc4.png]

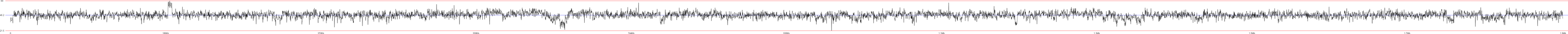

Supplement: Supplementary file 5 — Supplementary material [file mmc5.zip › mmc5.png]

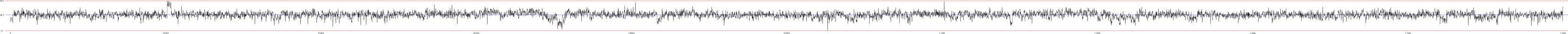

Supplement: Supplementary file 9 — Supplementary material [file mmc9.zip › mmc9.png]

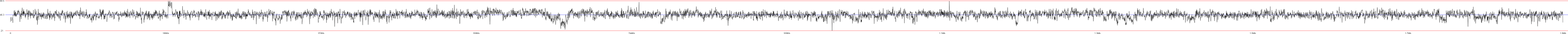

Supplement: Supplementary file 11 — Supplementary material [file mmc11.zip › mmc11.png]

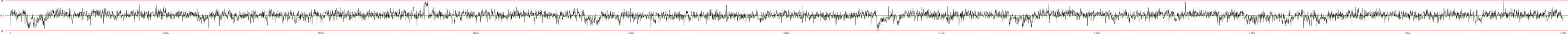

Supplement: Supplementary file 13 — Supplementary material [file mmc13.zip › mmc13.png]

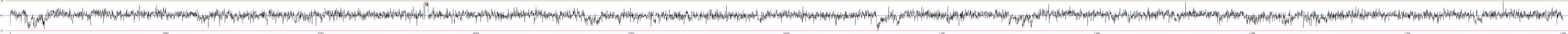

Supplement: Supplementary file 14 — Supplementary material [file mmc14.zip › mmc14.png]

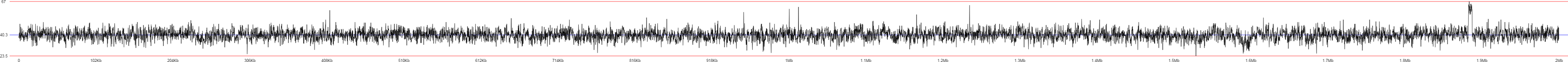

Supplement: Supplementary file 15 — Supplementary material [file mmc15.zip › mmc15.png]
